# ECE2 is a prognostic biomarker associated with m6A modification and involved in immune infiltration of lung adenocarcinoma

**DOI:** 10.3389/fendo.2022.1013238

**Published:** 2022-10-10

**Authors:** Yao-Hua Zhang, Jing Zeng, Xu-Sheng Liu, Yan Gao, Xue-Yan Kui, Xiao-Yu Liu, Yu Zhang, Zhi-Jun Pei

**Affiliations:** ^1^ Department of Nuclear Medicine, Taihe Hospital, Hubei University of Medicine, Shiyan, China; ^2^ Department of Infection Control, Taihe Hospital, Hubei University of Medicine, Shiyan, China; ^3^ Hubei Key Laboratory of Embryonic Stem Cell Research, Shiyan, China

**Keywords:** ECE2, lung adenocarcinoma, prognostic biomarker, immune infiltration, m6A modification

## Abstract

**Background:**

The targeted therapy for lung cancer relies on prognostic genes and requires further research. No research has been conducted to determine the effect of endothelin-converting enzyme 2 (ECE2) in lung cancer.

**Methods:**

We analyzed the expression of ECE2 in lung adenocarcinoma (LUAD) and normal adjacent tissues and its relationship with clinicopathological characteristics from The Cancer Genome Atlas (TCGA) and Gene Expression Omnibus database (GEO). Immunohistochemical staining was used to further validate the findings. GO/KEGG enrichment analysis and gene set enrichment analysis (GSEA) of ECE2 co-expression were performed using R software. Data from TIMER, the GEPIA database, and TCGA were analyzed to determine the relationship between ECE2 expression and LUAD immune infiltration. To investigate the relationship between ECE2 expression levels and LUAD m6A modification, TCGA data and GEO data were analyzed.

**Results:**

ECE2 is highly expressed in various cancers including LUAD. ECE2 showed high accuracy in distinguishing tumor and normal sample results. The expression level of ECE2 in LUAD was significantly correlated with tumor stage and prognosis. GO/KEGG enrichment analysis showed that ECE2 was closely related to mitochondrial gene expression, ATPase activity and cell cycle. GSEA analysis showed that ECE2-related differential gene enrichment pathways were related to mitotic cell cycle, MYC pathway, PLK1 pathway, DNA methylation pathway, HIF1A pathway and Oxidative stress-induced cellular senescence. Analysis of the TIMER, GEPIA database, and TCGA datasets showed that ECE2 expression levels were significantly negatively correlated with B cells, CD4+ cells, M2 macrophages, neutrophils, and dendritic cells. TCGA and GEO datasets showed that ECE2 was significantly associated with m6A modification-related genes HNRNPC, IGF2BP1, IGF2BP3 and RBM1.

**Conclusion:**

ECE2 is associated with m6A modification and immune infiltration and is a prognostic biomarker in LUAD.

## Introduction

Analysis of global data shows that lung cancer is the second most prevalent cancer among both sexes, accounting for about 18% of the estimated 9.9 million cancer-related deaths (1). Among lung cancers, 85% of the histological subtypes are non-small cell lung cancers, with lung adenocarcinoma being the most common (2). Multimodal treatment options for lung adenocarcinoma patients are tailored and vary based on cancer subtype, presenting status, age, tumor grade, tumor location, and molecular characteristics (3). Most LUAD patients will undergo surgery, molecular targeted therapy, immunotherapy, radiotherapy, and chemotherapy (4–6). Despite recent advances in disease diagnosis and cancer treatment, the prognosis for patients with intermediate and advanced LUAD remains poor. Therefore, it is extremely important to identify promising prognostic biomarkers for lung adenocarcinoma and develop new targets for the early diagnosis and treatment of tumors.

Endothelin-converting enzyme 2 (ECE2) may act as an intracellular processing enzyme (7). The name stems from its ability to convert the macromolecular endothelin into the powerful vasoactive peptide endothelin-1 (8). There are few existing studies on ECE2, but there are studies that identify ECE2 by bilevel selection method may have important prognostic significance for LUAD (9). However, no comprehensive biological function study of ECE2 in LUAD has been performed.

In this study, the Cancer Genome Atlas (TCGA) LUAD dataset and Gene Expression Omnibus (GEO) database was downloaded to analyze the potential value of ECE2 in LUAD. We investigated the differential expression of ECE2 in different tumor types based on bioinformatics using R software and online databases, and used immunohistochemical staining to verify the differential expression of ECE2 in LUAD tumors and adjacent tumors. The related genes of ECE2 were analyzed from various aspects. The biological functions and signal transduction of these genes were analyzed. Finally, the relationship between ECE2 expression level and immune cell infiltration and m6A was analyzed.

## Materials and methods

### Expression of ECE2 in LUAD

The TCGA_GTEx-LUAD database we used is the RNAseq data of TCGA and GTEx in TPM format uniformly processed by UCSC XENA through the Toil process, and used to analyze the expression differences of ECE2 in different tumors (n=15776) (10). The TCGA-LUAD database (https://portal.gdc.cancer.gov/) (11) was used to analyze differences in ECE2 expression between normal tissues and LUAD tumor tissues(n = 594). We downloaded the GSE31210, GSE116959, GSE32863 and GSE75037 datasets from the GEO database (www.ncbi.nlm.nih.gov/geo) to analyze differences in ECE2 expression between LUAD and normal samples. Immunohistochemical staining was used to confirm the difference in ECE2 protein expression between LUAD tumor tissues and adjacent tissues. ROC curves and KM curves were used to estimate the prognosis and diagnostic value of ECE2 in LUAD patients. Moreover, we examined the relationship between ECE2 expression levels and clinicopathological features among LUAD patients using the TCGA LUAD dataset.

### Lung cancer tissue samples

We recruited lung cancer patients undergoing surgery at Taihe Hospital in Shiyan City, Hubei Province. This study was approved by the Medical Ethics Committee of Taihe Hospital and followed the Declaration of Helsinki and its subsequent amendments (NO.2022KS010).

### Immunohistochemistry staining

For IHC staining, sections were deparaffinized in xylene and rehydrated in alcohol, then endogenous peroxidase was inactivated by treatment with 3% H_2_O_2_ for 5 min. Then, antigen retrieval was performed in a pressure cooker with sodium citrate buffer (pH 6.0). After blocking with 5% goat serum for 30 min at room temperature, sections were incubated with rabbit ECE2 monoclonal antibody (1:200, Abcam, USA) overnight in a 4°C refrigerator. Sections were coupled with goat anti-mouse IgG-HRP secondary antibody (1:400, Abcam, USA) for 1 hour at room temperature, then each incubated section was stained with DAB reagent and finally counterstained with hematoxylin (12).

### LUAD enrichment analysis of the gene co-expression network for ECE2

The TCGA LUAD dataset was analyzed using the stat package of the R software to investigate co-expressed genes associated with ECE2 expression. Calculate the spearman correlation coefficient to test statistical correlation, and use the ggplot2 package of R software to draw volcano plots and heat maps for display, where the critical value is set to 0.3, p<0.05 was considered statistically significant. Gene Ontology (GO) functional and Kyoto Encyclopedia of Genes and Genomes (KEGG) pathway enrichment analyses were performed on co-expressed genes by the clusterProfiler package (version: 3.6.3) (13) of the R software, visual analysis of data was performed by ggplot2 software package.

### Gene set enrichment analysis

To further understand the underlying mechanism of ECE2, based on the median expression of ECE2, we divided the dataset from TCGA-LUAD into high and low expression groups horizontally, and performed single-gene differential analysis using the DESeq2 [version 1.26.0] package (14). All differential genes were subjected to GSEA (www.gsea-msigdb.org/gsea/index.jsp) (15)analysis with the clusterProfiler package [version 3.14.3] (13)to investigate whether these genes are enriched in meaningful biological processes. The reference gene set is c2.cp.v7.2.symbols.gmt [Curated], and the ggplot2 package [version 3.3.3] visualizes enriched pathways that satisfy FDR (qvalue) < 0.25, P < 0.05.

### Tissue immune infiltrating cells and ECE2

To further explore the potential immunoregulatory mechanism of ECE2 in the regulation of tumor-infiltrating immune cells, we used the TIMER database (www.cistrome.shinyapps.io/TIMER) (16, 17) to evaluate the correlation between ECE2 expression and immune-infiltrating cells in TCGA LUAD samples. Immune infiltrating cells included tumor purity, B cells, neutrophils, CD4+ T cells, macrophages, CD8+ T cells, and dendritic cells. We analyzed the relationship between ECE2 copy number variation (CNV) and immune cell infiltration using the somatic copy number alteration (SCNA) module from the TIMER database. Using the built-in algorithm ssGSEA of the GSVA package (18), the expression differences of 24 immune cells in the LUAD samples of the ECE2 high and low expression groups were analyzed, including T cells, aDC (activated DC), B cells, CD8 T cells, Cytotoxic cells, DC, Eosinophils, iDC (immature DC), Macrophages, Mast cells, Neutrophils, NK CD56bright cells, NK CD56dim cells, NK cells, pDC (Plasmacytoid DC), T helper cells, Tcm (T central memory), Tem (T effector memory), Tfh (T follicular helper), Tgd (T gamma delta), Th1 cells, Th17 cells, Th2 cells and Treg (19).The Kaplan-Meier curve was used to show the relationship between the degree of immune cell infiltration and the prognosis of LUAD patients, as well as the relationship between the ECE2 expression and the prognosis of LUAD patients. Furthermore, we analyzed the association between ECE2 and immune cell marker genes in LUAD samples from three databases TIMER, GEPIA and TCGA.

### ECE2 expression and m6A modification in LUAD

The correlation between ECE2 expression and m6A-related gene expression in GSE31210 and TCGA LUAD datasets was analyzed using the R package, Including ZC3H13, YTHDF3, HNRNPA2B1, IGF2BP1, IGF2BP3, YTHDC2, YTHDF1, FTO, HNRNPC, METTL14, METTL3, WTAP, RBM15, ALKBH5, IGF2BP2, RBMX, RBM15B, YTHDC1, VIRMA, and YTHDF2 (20). The prognostic value of these 20 m6A-associated genes in LUAD samples was analyzed using GEPIA 2.0(http://gepia2.cancer-pku.cn/#index) (21). Data were visualized and analyzed by the ggplot2 software package.

### Statistical analysis

Most of the research in this paper was done using the bioinformatics tools mentioned above and the Xiantao platform (www.xiantao.love). Continuous variables were expressed as mean ± standard deviation or median and interquartile range, and comparisons between groups were performed using Student’s t-test or Wilcoxon test. Categorical variables were expressed as counts and percentages, and comparisons between groups were performed using the chi-square test or Fisher exact probability method. P<0.05 was considered statistically significant.

## Results

### ECE2 expression in patients with LUAD

**Figure 1:**
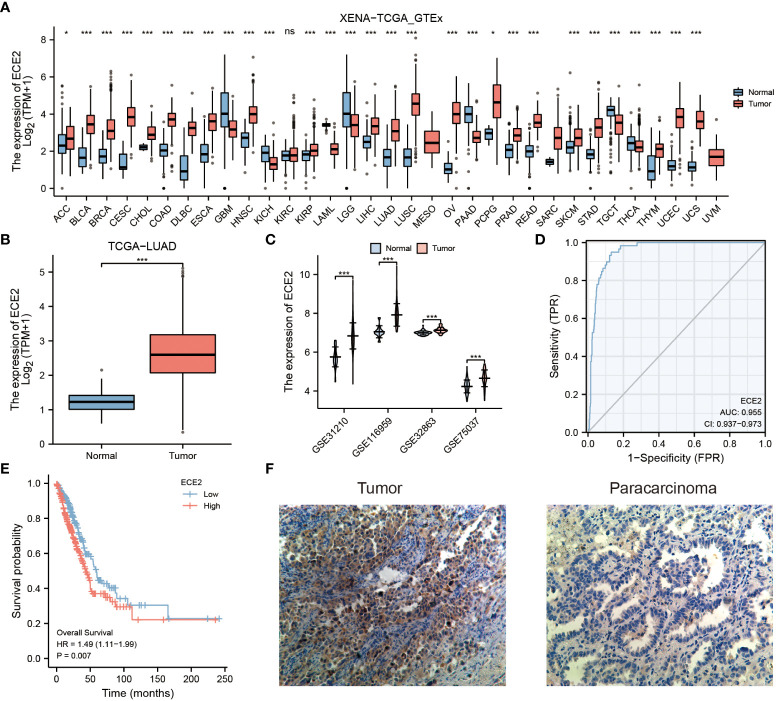
The expression of ECE2 in lung adenocarcinoma (LUAD) and pan-carcinoma. **(A)** The expression level of ECE2 in different tumors was evaluated with the XENA-TCGA_GTEx dataset (Wilcoxon rank sum test). **(B)** Boxplots of ECE2 expression between LUAD and normal tissue in the TCGA dataset (Wilcoxon rank sum test). **(C)** Differences in ECE2 expression between LUAD and normal tissues in GSE31210, GSE116959, GSE32863, and GSE70537 datasets (Wilcoxon rank sum test). **(D)** ROC curve analysis of ECE2 diagnosis. **(E)** The survival curve of ECE2. **(F)** Immunohistochemical analysis of ECE2 expression in LUAD tumor and para-cancer. *P < 0.05; ***P < 0.001. ns, not significant.

We analyzed ECE2 mRNA expression in different tumors using the XENA-TCGA_GTEx dataset. **Figure 1A** shows the differences in ECE2 expression between different tumor types and normal tissues. ECE2 in glioblastoma multiforme (GBM), renal chromophore (KICH), acute myeloid leukemia (LAML) and brain low-grade glioma (LGG), pancreatic cancer (PAAD), testicular germ cell tumor (TGCT) and decreased expression in thyroid carcinoma (THCA). The expression level of ECE2 is significantly increased in different cancer tissues, including adrenocortical carcinoma (ACC), bladder urothelial carcinoma (BLCA), breast invasive carcinoma (BRCA), cervical squamous cell carcinoma and adenocarcinoma (CESC), cholangiocarcinoma (CHOL), colon adenocarcinoma (COAD), diffuse large B lymphoid neoplastic cell lymphoma (DLBC), esophageal cancer (ESCA), head and neck squamous cell carcinoma (HNSC), renal clear cell carcinoma (KIRC), renal papillary cell carcinoma (KIRP), hepatocellular carcinoma (LIHC), lung adenocarcinoma (LUAD), lung squamous cell carcinoma (LUSC), ovarian serous cystadenocarcinoma (OV), pheochromocytoma, and paraganglia tumor (PCPG), prostate cancer (PRAD), rectal adenocarcinoma (READ), cutaneous melanoma (SKCM), gastric adenocarcinoma (STAD), THYM, endometrial carcinoma (UCEC) and uterine carcinosarcoma (UCS). In the TCGA dataset, ECE2 mRNA levels were significantly higher in LUAD samples than in control normal samples (**Figure 1B**). Analysis of the GSE31210, GSE116959, GSE32863, and GSE75037 datasets showed that the expression level of ECE2 was significantly increased in the LUAD samples (**Figure 1C**). According to ROC analysis, in LUAD, ECE2 may be a good predictor of LUAD with an area under the curve of 0.955 (95% confidence interval: 0.937-0.973) (**Figure 1D**). Survival analysis showed that high expression of ECE2 in LUAD predicted poor survival. (HR = 1.49 (1.11-1.99), P = 0.007) (**Figure 1E**). Immunohistochemical analysis revealed that LUAD tumor tissue had significantly higher ECE2 protein levels than adjacent normal tissue (**Figure 1F**). Collectively, ECE2 mRNA and protein levels were elevated in LUAD tissues and could serve as prognostic biomarkers.

### ECE2 expression and LUAD patients’ clinicopathological parameters

**Figure 2:**
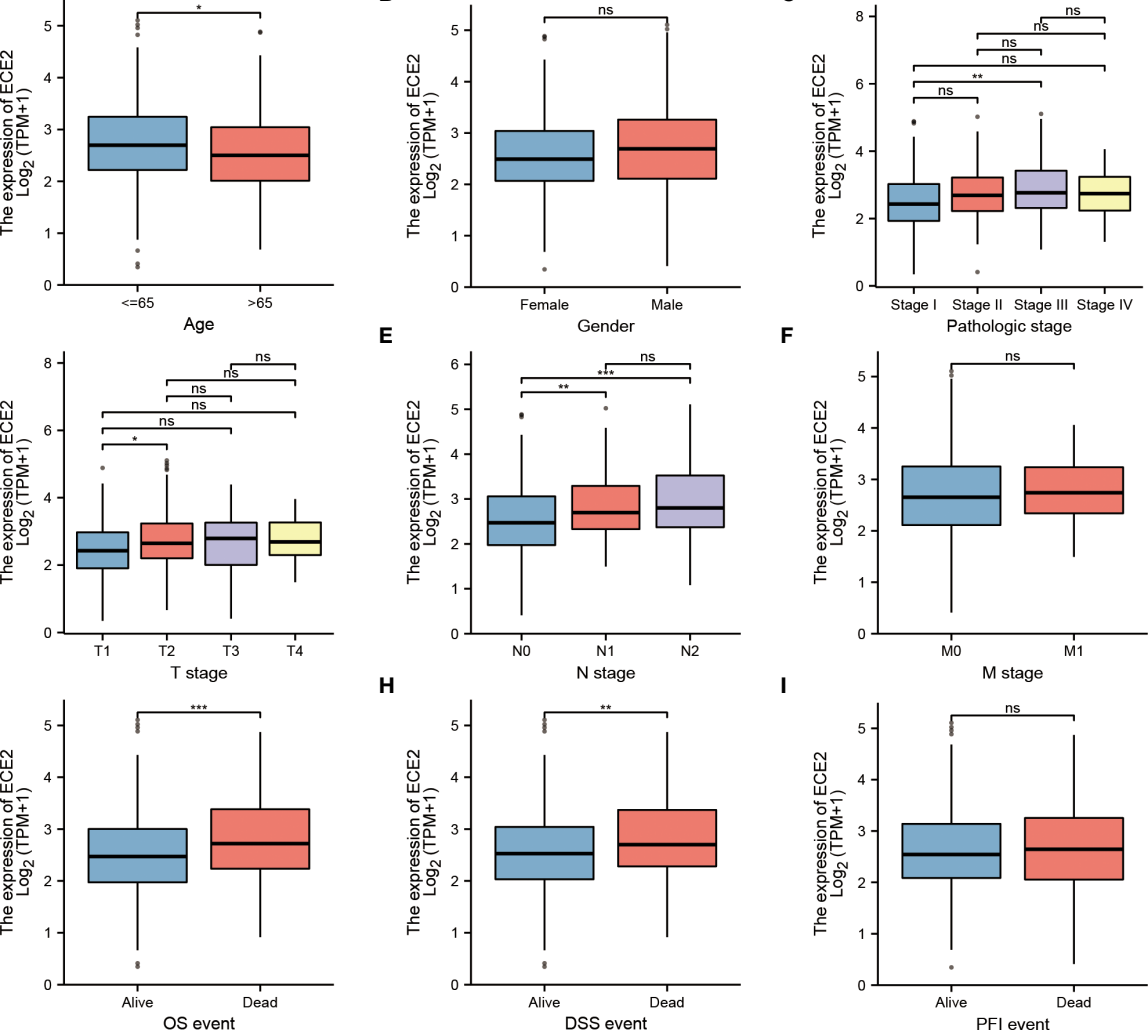
Relationship between ECE2 expression and clinicopathological parameters in patients with LUAD. **(A)** age (Wilcoxon rank sum test), **(B)** gender (Wilcoxon rank sum test), **(C)** pathologic stage (One-way ANOVA test), **(D)** T stage (Kruskal-Wallis Test), **(E)** N stage (Kruskal-Wallis Test), **(F)** M stage (Wilcoxon rank sum test), **(G)** OS event (Wilcoxon rank sum test), **(H)** DSS event (Wilcoxon rank sum test), **(I)** PFI event (Wilcoxon rank sum test). (Wilcoxon rank sum test) *P < 0.05; **P < 0.01; ***P < 0.001. ns, not significant.

Since the role of ECE2 in LUAD is still unclear, the correlation between ECE2 expression level and clinicopathological features can help determine the potential significance of ECE2 in LUAD development. We analyzed clinical sample (n=594) results from TCGA LUAD to investigate the importance of ECE2 in the clinical setting. The expression level of ECE2 in clinical patients aged <65 years was higher than that in clinical patients aged >65 years. The expression of ECE2 in stage III group was higher than that in stage I group, the expression of ECE2 in T1 group was lower than that in T2 group, and the expression of ECE2 in N1 and N2 groups was higher than that in N0 group. Deceased patients had significantly higher ECE2 expression levels than surviving patients in both OS and DSS events (**Figure 2**). **Table 1** summarizes the clinical features of ECE2 in LUAD in detail.

**Table 1:** Clinical characteristics of ECE2 in lung adenocarcinoma.

Characteristic	Low expression of ECE2	High expression of ECE2	p	Method
n	267	268		
T stage, n (%)			0.132	Chisq.test
T1	99 (18.6%)	76 (14.3%)		
T2	137 (25.8%)	152 (28.6%)		
T3	22 (4.1%)	27 (5.1%)		
T4	7 (1.3%)	12 (2.3%)		
N stage, n (%)			< 0.001	Fisher.test
N0	191 (36.8%)	157 (30.3%)		
N1	41 (7.9%)	54 (10.4%)		
N2	23 (4.4%)	51 (9.8%)		
N3	1 (0.2%)	1 (0.2%)		
M stage, n (%)			0.371	Chisq.test
M0	171 (44.3%)	190 (49.2%)		
M1	9 (2.3%)	16 (4.1%)		
Pathologic stage, n (%)			0.002	Chisq.test
Stage I	167 (31.7%)	127 (24.1%)		
Stage II	57 (10.8%)	66 (12.5%)		
Stage III	30 (5.7%)	54 (10.2%)		
Stage IV	10 (1.9%)	16 (3%)		
Gender, n (%)			0.062	Chisq.test
Female	154 (28.8%)	132 (24.7%)		
Male	113 (21.1%)	136 (25.4%)		
Age, n (%)			0.134	Chisq.test
<=65	117 (22.7%)	138 (26.7%)		
>65	138 (26.7%)	123 (23.8%)		
OS event, n (%)			0.010	Chisq.test
Alive	186 (34.8%)	157 (29.3%)		
Dead	81 (15.1%)	111 (20.7%)		
DSS event, n (%)			0.056	Chisq.test
Alive	198 (39.7%)	181 (36.3%)		
Dead	50 (10%)	70 (14%)		
PFI event, n (%)			0.453	Chisq.test
Alive	159 (29.7%)	150 (28%)		
Dead	108 (20.2%)	118 (22.1%)		
Age, median (IQR)	67 (60, 73)	65 (58, 71)	0.018	Wilcoxon

### LUAD Co-expression network analysisof ECE2

**Figure 3:**
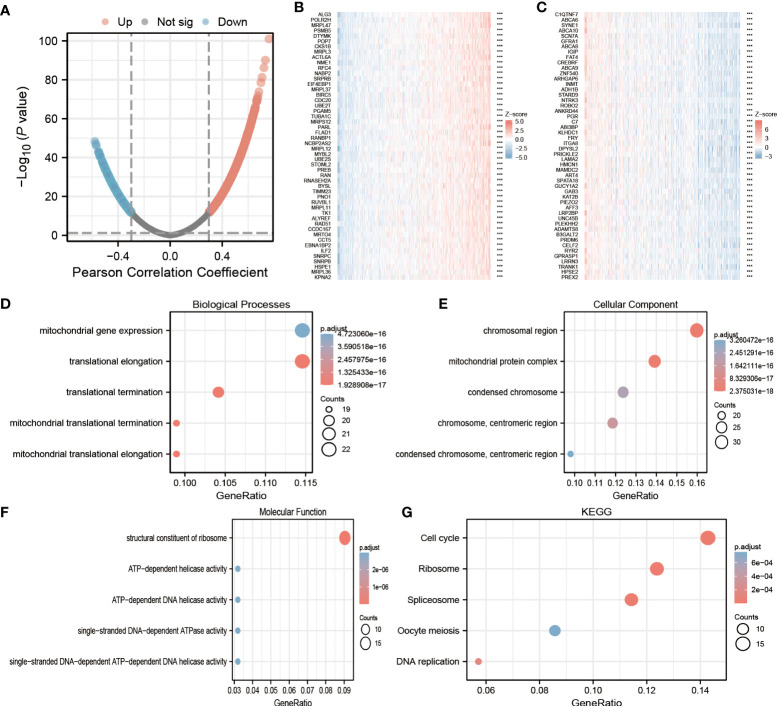
Lung adenocarcinoma (LUAD) enrichment analysis of ECE2 functional networks. **(A)** The volcano map showed co-expression genes associated with ECE2 expression in TCGA LUAD data sets. **(B, C)** Heat maps showing the top 50 co-expression genes positively and negatively correlated with ECE2 expression in the LUAD data sets. **(D–F)** Enrichment analysis of gene ontology (GO) terms for ECE2 co-expression genes. **(G)** Enrichment analysis of Kyoto Encyclopedia of Genes and Genomes (KEGG) terms for ECE2 co-expression genes. ***P < 0.001.

To predict the biological role of ECE2 in LUAD, we analyzed the co-expressed genes associated with ECE2 using the statistical package of R software. In which only data for protein-coding genes are retained. According to **Figure 3A**, ECE2 was positively correlated with 6196 genes, whereas 7070 genes were negatively correlated with it. The heatmap mainly shows the top 50 genes positively and negatively correlated with ECE2 (**Figures 3B, C**).

By using the R software package, GO functional enrichment and KEGG pathway enrichment analysis were performed on the top 200 co-expressed genes positively correlated with ECE2 expression. Under the condition of P. adj < 0.05 & qvalue < 0.2, ECE2 co-expressed genes with 407 biological processes (Go-BP), 103 cellular components (GO-CC), 25 molecular functions (GO-MF) and 12 KEGG correlation. The bubble chart shows the top 5 messages from GO-BP, GO-CC, GO-MF and KEGG. Based on functional annotation of GO, ECE2 co-expressed genes were mainly involved in mitochondrial gene expression, chromosomal regions and ATPase activity (**Figures 3D–F**). In KEGG pathway analysis, ECE2 co-expression was mainly associated with meiosis, DNA replication, spliceosome, ribosome and cell cycle (**Figure 3G**).

### Gene set enrichment analysis

**Figure 4:**
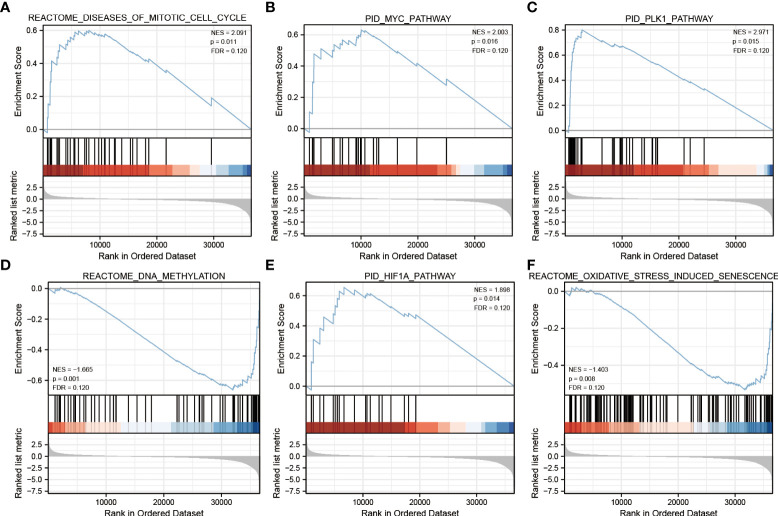
Gene Set Enrichment Analysis. **(A)** mitotic cell cycle **(B)** MYC pathway **(C)** PLK1 pathway **(D)** DNA methylation pathway **(E)** HIF1A pathway **(F)** Oxidative stress-induced cellular senescence.

To explore the potential function of ECE2 in LUAD, we performed GSEA enrichment for differential genes of ECE2. A total of 486 genomes were identified, including mitotic cell cycle (FDR=0.120, P=0.011), MYC pathway (FDR=0.120, P=0.016), PLK1 pathway (FDR=0.120, P=0.015), DNA methylation pathway (FDR=0.120, P=0.001), HIF1A pathway (FDR=0.120, P=0.014) and Oxidative stress-induced cellular senescence (FDR=0.120, P=0.008) (**Figure 4**). **Table 2** provides detailed information.

**Table 2:** Gene sets enriched in correlated with ECE2 mRNA expression phenotype.

ID	ES	NES	P value
REACTOME_DISEASES_OF_MITOTIC_CELL_CYCLE	0.632	2.091	0.011
PID_MYC_PATHWAY	0.655	2.003	0.016
PID_PLK1_PATHWAY	0.801	2.971	0.015
REACTOME_DNA_METHYLATION	-0.663	-1.665	0.001
PID HIF1A_PATHWAY	0.655	1.898	0.014
REACTOME_OXIDATIVE_STRESS_INDUCED_SENESCENCE	-0.534	-1.403	0.008

### Tissue immune infiltrating cells and ECE2

**Figure 5:**
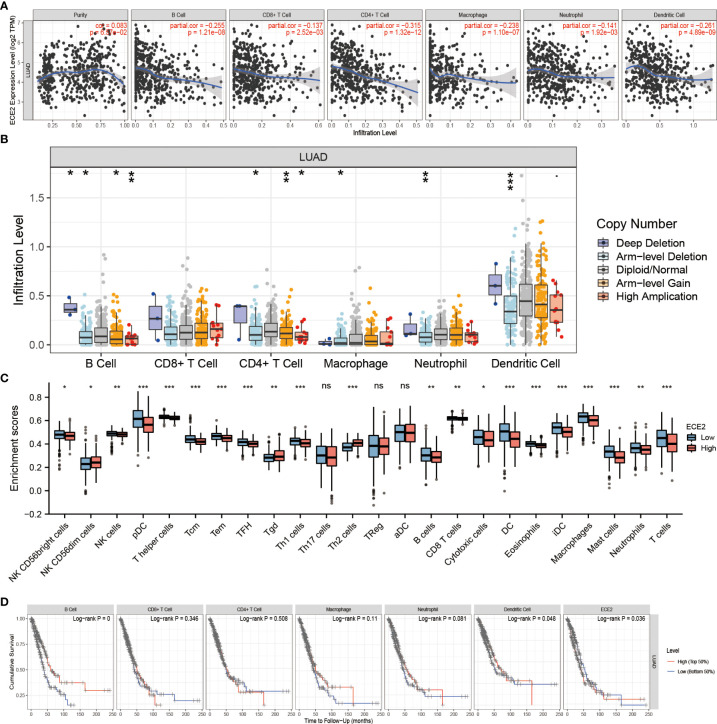
Correlation between ECE2 and Tumor Immune Infiltrating Cells. **(A)** Correlation between ECE2 expression and immune infiltrating cells in LUAD (Spearman correlation analysis). **(B)** ECE2 CNV affects the infiltrating levels of B cell, CD4+ T cell, macrophages, neutrophils, and dendritic cells in LUAD. **(C)** Changes in 24 immune cell subtypes between high and low ECE2 expression groups in LUAD tumor samples (Wilcoxon rank sum test). **(D)** Kaplan-Meier plots of immune infiltration and ECE2 expression levels in LUAD. (Logrank test) *P < 0.05; **P < 0.01; ***P < 0.001. ns, not significant.

To investigate the role of ECE2 in tumor immunity, we determined the correlation between ECE2 expression and immune-infiltrating cells in LUAD using the TIMER database. **Figure 5A** demonstrates that the expression of ECE2 was negatively correlated with B cells (r=-0.255, P=1.21E-08), CD4 T cells (R =-0.315, P=1.32E-12), CD8 T cells (R =-0.137, P=0.003), neutrophils (R =-0.141, P=0.002), dendritic cells (R =-0.261, P=4.89E-09), and macrophages (r=-0.238, P=1.10E-07). The action of ECE2 with different copy numbers significantly changed the level of immune infiltration in LUAD, as shown in **Figure 5B**. The results of R package analysis showed that ECE2 expression levels were correlated with tumor immune infiltrating cells, including NK CD56bright cells (P = 0.02), NK CD56dim cells (P = 0.043), NK cells (P = 0.001), pDC (P < 0.001), T helper cells (P < 0.001), Tcm (P < 0.001), Tem (P < 0.001), TFH (P < 0.001), Tgd (P = 0.009), Th1 cells (P < 0.001), Th2 cells (P < 0.001), B cells (p=0.003),CD8 T cells (p=0.007), Cytotoxic cells (p=0.02), DC (P < 0.001), Eosinophils (P < 0.001), iDC (P < 0.001), Macrophages (P < 0.001), Mast cells (P < 0.001), Neutrophils (p=0.009) and T cells (P < 0.001)(**Figure 5C**). Kaplan-Meier curves showed that B cells (P=0.0002), dendritic cells (P=0.0475), and ECE2 expression (P=0.036) were associated with prognosis in LUAD (**Figure 5D**). These findings highlight the important role ECE2 plays in the immune infiltration of dendritic cells and B cells.

**Figure 6:**
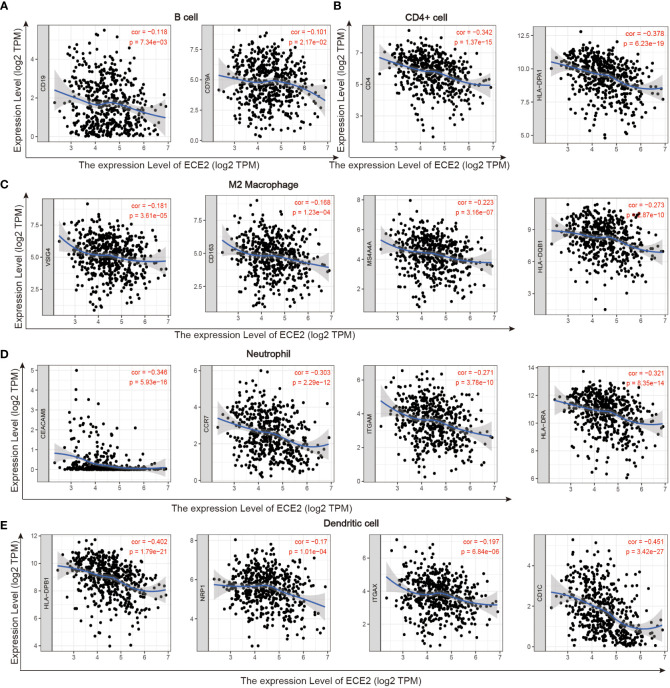
TIMER database correlation analysis of ECE2 expression with marker genes of infiltrating immune cells in LUAD. **(A)** B cell **(B)** CD4+ cell **(C)** M2 Macrophage **(D)** Neutrophil and **(E)** Dendritic cell.

The role of ECE2 in tumor immunity remains to be further explored, and we used GEPIA, TIMER and TCGA databases to study the correlation between ECE2 expression levels in LUAD and several markers of immune infiltration. ECE2 expression was significantly negatively correlated with B cell biomarkers (CD19 and CD79A) and T cell biomarkers (CD4), M2 macrophage biomarkers (CD163, VSIG4, and MS4A4A), neutrophil biomarkers (CEACAM8, ITGAM and CCR7) and dendritic cell biomarkers (HLA-DPB1, HLA-DQB1, HLA-DRA, HLA-DPA1, CD1C, NRP1, and ITGAX) (**Figure 6** and **Table 3**). These findings support a negative correlation between ECE2 and immune cell infiltration.

**Table 3:** Correlation analysis between ECE2 and lung adenocarcinoma immune cell biomarkers measured by GEPIA, TIMER and TCGA databases (Spearman correlation test).

Immune cell	Biomarker	GEPIA		TIMER		TCGA	
		R	P	R	P	R	P
**B cell**	CD19	-0.180	**5.7E-05**	−0.118	**7.34E−03**	−0.125	**4.00E-03**
	CD79A	-0.190	**2.3E-05**	−0.101	**2.17E−02**	−0.093	**3.10E-02**
CD8+ T cell	CD8A	-0.080	7.80E-02	−0.041	3.55E−01	−0.007	8.71E-01
	CD8B	−0.02	6.70E-01	0.017	7.01E−01	0.046	2.90E-01
**CD4+ T cell**	CD4	-0.350	**3.6E-15**	−0.342	**1.37E-15**	−0.257	**<0.001**
M1 macrophage	NOS2	-0.034	4.60E-01	−0.042	3.41E-01	0.019	6.67E-01
	IRF5	0.460	9.80E-02	−0.101	**2.12E-02**	−0.009	8.36E-01
	PTGS2	0.080	8.00E-02	0.078	7.58E-02	0.054	2.10E-01
**M2 macrophage**	CD163	-0.120	**6.00E-03**	−0.168	**1.23E-04**	−0.150	**<0.001**
	VSIG4	-0.180	**8.20E-05**	−0.181	**3.61E-05**	−0.094	**2.90E-02**
	MS4A4A	-0.220	**8.50E-07**	−0.223	**3.16E-07**	−0.157	**<0.001**
**Neutrophil**	CEACAM8	-0.320	**3.5E-13**	−0.346	**5.93E-16**	−0.347	**<0.001**
	ITGAM	-0.290	**1.10E-10**	−0.271	**3.78E−10**	−0.253	**<0.001**
	CCR7	-0.330	**5.2E−14**	−0.303	**2.29E−12**	−0.283	**<0.001**
**Dendritic cell**	HLA-DPB1	-0.390	**5.2E−19**	−0.402	**1.79E−21**	−0.282	**<0.001**
	HLA-DQB1	-0.210	**4.2E−06**	−0.273	**2.87E−10**	−0.204	**< 0.001**
	HLA-DRA	-0.320	**6.2E−13**	−0.321	**8.35E−14**	−0.196	**< 0.001**
	HLA-DPA1	-0.370	**6.1E−17**	−0.378	**6.23E-19**	−0.254	**< 0.001**
	CD1C	-0.430	**3.4E−23**	−0.451	**3.42E-27**	−0.370	**< 0.001**
	NRP1	-0.150	**7.80E-04**	−0.17	**1.01E−04**	−0.184	**< 0.001**
	ITGAX	-0.200	**1.5E-05**	−0.197	**6.84E−06**	−0.203	**< 0.001**

The bold part indicates that the correlation between the expression level of ECE2 and immune cell markers in LUAD was statistically significant (P<0.05).

### ECE2 expression and m6A modification in LUAD

**Figure 7:**
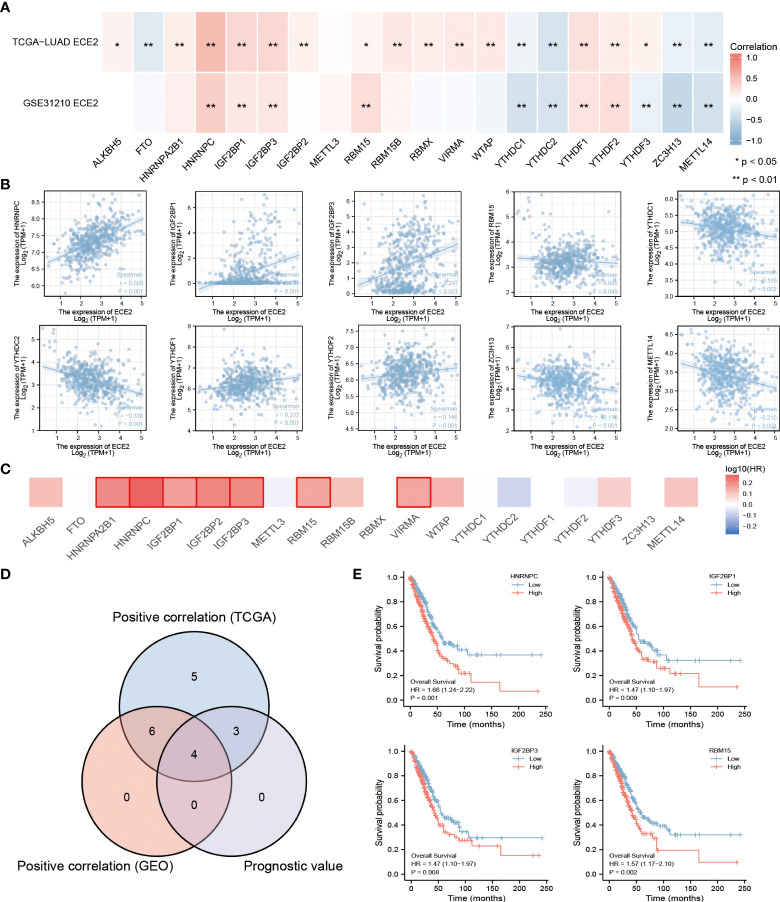
An analysis of the correlation between ECE2 expression and m6A-related genes in lung adenocarcinoma (LUAD). **(A)** We examined the correlation between the expression of ECE2 and m6A-related genes in LUAD using GSE31210 and the TCGA LUAD data sets. **(B)** To illustrate the correlation between ECE2 and m6A gene expression, scatterplots were drawn. **(C)** Survival maps for the m6A-related gene. **(D)** Venn diagrams show their overlapping genes.**(E)** Kaplan-Meier curve of HNRNPC, IGH2BP1, IGH2BP3 and RBM15. (Wilcoxon rank sum test) *P < 0.05; **P < 0.01.

By comparing TCGA LUAD and GSE31210 data sets, In LUAD, we examined the correlation between ECE2 expression and 20 m6A-related genes. TCGA LUAD data showed that ECE2 expression was significantly positively correlated with ALKBH5, HNRNPA2B1, HNRNPC, IGF2BP1, IGF2BP3, IGF2BP2, RBM15, RBM15B, RBMX, VIRMA, WTAP, YTHDF1, YTHDF2 and YTHDF3, whereas ECE2 expression level was significantly correlated with FTO, YTHDC1, YTHDC2, ZC3H13 and METTL14 were significantly negatively correlated. The GSE31210 data showed that ECE2 expression was significantly positively correlated with HNRNPC, IGF2BP1, IGF2BP3, RBM15, YTHDF1 and YTHDF2, whereas ECE2 expression was significantly negatively correlated with YTHDC1, YTHDC2, YTHDF3, ZC3H13 and METTL14 (**Figure 7A**). The scatter plot shows the correlation between ECE2 and M6A-related genes (**Figure 7B**).

We showed the prognostic value of these 20 m6A-related genes in LUAD using a survival map (**Figure 7C**). We demonstrated the intersection of related genes with prognostic genes using a Wayne diagram (**Figure 7D**). Survival analysis plots for the above crossover genes are shown with Kaplan-Meier curves. The results showed that the high expression of HNRNPC(p=0.001), IGF2BP1(p=0.009), IGF2BP3 (p=0.008) and RBM15 (p=0.002) was associated with poor prognosis in LUAD. These results indicate that ECE2 may be closely related to the m6A of LUAD, especially through its regulation with HNRNPC may influence the progression and prognosis of LUAD (**Figure 7E**).

## Discussion

There is no doubt that LUAD is one of the most prevalent malignant tumors in humans. Due to the lack of clinical characteristics of patients with early LUAD, a large number of patients develop metastases (22, 23). Therefore, the identification of new molecular targets and molecular pathways is useful for providing new insights into the treatment and prognosis of LUAD. Previous research has demonstrated that all four subtypes of ECE2 are located in endocrine vesicles and capable of degrading intracellular neuropeptides under mildly acidic conditions (24). ECE2 is mainly expressed in neural tissues (8). The ECE2 gene coding variant can be used to identify Alzheimer’s disease (25). ECE2 regulates neurogenesis and neuronal migration during human cortical development (26). There are currently few investigations on ECE2, and its role in other cancers including LUAD remains unknown. Consequently, it is crucial to investigate the role of ECE2 in LUAD. Our study found that high expression of ECE2 predicts poor prognosis in LUAD, suggesting that perhaps we can improve the prognosis of patients with LUAD by altering the expression level of ECE2 in LUAD.

In this study, based on the XENA-TCGA_GTEx, TCGA and GEO datasets, we found that ECE2 is highly expressed in various tumors including LUAD. The conclusion that ECE2 is highly expressed in LUAD was further confirmed by immunohistochemical staining. We also found that the high expression level of ECE2 has high accuracy in the diagnosis of LUAD and is closely related to the poor prognosis and clinical characteristics of LUAD patients.

Predicting the underlying mechanism by which ECE2 regulates the development of LUAD is one of the major contributions of this study. GO/KEGG enrichment analysis indicated that the co-expressed genes of ECE2 were involved in mitochondrial expression, ATPase activity and cell cycle function. Some studies have found that the above-mentioned biological functions play an important role in the occurrence and development of tumors (27–29). These findings suggest that the co-expression network of ECE2 plays a key role in tumor initiation and progression. The enrichment analysis of the GSEA pathway showed that differential genes of ECE2 were enriched in the mitotic cell cycle, MYC pathway, PLK1 pathway, DNA methylation pathway, HIF1A pathway and Oxidative stress-induced cellular senescence pathways. The proto-oncogene MYC has been implicated in the formation, maintenance, and progression of a number of different cancer types (30–32). As a transcriptional regulator, MYC protein mainly plays a role in regulating cell growth, cell cycle, differentiation, apoptosis and metabolism (33). At present, a large number of literature studies have shown that MYC is closely related to the occurrence and development of LUAD (34–37). However, whether ECE2 can affect the cell cycle and regulate the development of LUAD by regulating the expression of MYC is unclear. PLK1 is the most widely studied member of the PLK kinase family and is primarily involved in cell cycle regulation and cancer development (38, 39). DNA methylation as a novel therapeutic target plays a key role in the pathogenesis of lung cancer (40). We speculate that ECE2 may affect the development of LUAD by participating in the methylation pathway. Hypoxia is known to be a driver of glycolysis. HIF1A plays an important role in glycolysis as it can induce the expression of glycolytic enzymes to generate ATP (41). Oxidative stress has also been extensively studied in recent years as an important factor regulating tumor development and response to anticancer therapy (42, 43). These results suggest that ECE2 may play a role in LUAD by affecting these signaling pathways, thereby contributing to the poor prognosis of LUAD. However, the specific regulatory mechanism needs to be further explored.

Investigating the correlation between ECE2 and different immune cell infiltration in LUAD is also an important aspect of this study. The immune infiltration of tumor cells is closely related to the treatment and prognosis of cancer (44, 45). Our results showed that the expression level of ECE2 in LUAD was negatively correlated with most immune cells, including B Cell, CD8+ T Cell, CD4+ T Cell, Macrophage, Neutrophil and Dendritic Cell. In addition, the expression of various immune cells differed in LUAD with different ECE2 expression. Additionally, we found B cells and DCs to be factors associated with cumulative survival of LUAD over time. These results suggest that ECE2 may be involved in the immune response in LUAD. B cells are important immune cells in the human body and they play an important role in anti-tumor defense (46, 47). B cells can also be used as an immune checkpoint for LUAD, which improves the diagnosis and treatment rates for LUAD patients (48). Dendritic cells are the most efficient antigen-presenting cells and play an important role in T cell-mediated immune response (49, 50). It is increasingly recognized that the presence and function of Dendritic Cells in the tumor microenvironment (TME) may determine the efficacy of cancer immunotherapy as well as conventional cancer therapies, and is the right choice for cancer immunotherapy (51). It is suggested that ECE2 may have potential effects on tumor immunology.

In recent years, with the rapid development of tumor immunotherapy, people are increasingly aware of the role of tumor in immune cells in the occurrence and development of cancer. Finding related genes in the tumor microenvironment can be used as diagnostic and prognostic markers or as therapeutic targets (52). In the correlation analysis with different immune marker genes, it was found that ECE2 was significantly negatively correlated with immune marker genes. In this study, the expression levels of ECE2 and some immune marker genes were consistent with the expression levels of ECE2 in immune cells. The relationship between different immune marker genes and ECE2 expression implicates the importance of ECE2 in regulating the LUAD tumor immune microenvironment. These findings suggest that tumor immune infiltration may partially explain ECE2-mediated LUAD development. Our results provide initial guidance on the targets of immune infiltration in LUAD.

M6A modification, as a part of methylation modification, can affect the occurrence and development of cancer by regulating various biological functions of cancer (53–56). And some studies have found that the expression pattern of m6A regulators may help to formulate effective treatment strategies for LUAD (57). In the present study, we found that the expression of ECE2 was significantly correlated with HNRNPC, IGF2BP1, IGF2BP3 and RBM15. As a newly discovered m6A recognition reader protein (56), HNRNPC is responsible for recognizing m6A modification groups in the nucleus, mediating alternative splicing of mRNA precursors to regulate the abundance of targets, and can specifically bind to p53 (58). Overexpression of IGF2BP1 stabilizes PEG10 mRNA in an m6A-dependent manner and promotes endometrial cancer progression (59). In addition, several studies have shown that IGF2BP3 plays an important role in the occurrence and development of LUAD (60–62). RBM15 is an RNA-binding protein that plays an important role in cell growth and apoptosis by regulating various signaling pathways, including Notch and Wnt (63). Knockdown of RBM15 suppressed the proliferation and metastasis of colon cancer cells (64). We believe that the cancer-promoting role of ECE2 gene is related to the modification of m6A, which may affect the methylation level of LUAD, especially HNRNPC, through its association with HNRNPC, IGF2BP1, IGF2BP3 or RBM15, and ultimately affect the progression of LUAD.

This paper is the first to investigate the relationship of ECE2 in LUAD with immune infiltration, methylation and patient prognosis. In terms of immune infiltration, the expression level of ECE2 is negatively correlated with B cells and dendritic cells, which may affect tumor immunity through B cells and dendritic cells. In terms of methylation, the expression level of ECE2 was significantly negatively correlated with HNRNPC, IGF2BP1, IGF2BP3 and RBM15, which may affect the tumor progression of LUAD by affecting the m6A methylation level. ECE2 may be a prognostic biomarker marker for LUAD. However, the current study is based on bioinformatics analysis, so follow-up experimental studies are needed to validate these findings.

## Data availability statement

The original contributions presented in the study are included in the article/supplementary material. Further inquiries can be directed to the corresponding author.

## Ethics statement

The studies involving human participants were reviewed and approved by Ethics Committee of Taihe Hospital Affiliated of Hubei University of Medicine. Written informed consent for participation was not required for this study in accordance with the national legislation and the institutional requirements.

## Author contributions

Z-JP conceived and designed the study. YG and Z-JP as well as acquisition and management funds. Y-HZ, JZ, X-SL, YG, X-YK, X-YL, and YZ captured, collected, and extracted the data contained in this analysis. JZ, Y-HZ and X-SL analysis data. Y-HZ originally drafted the paper. Final draft read and approved by all authors.

## Funding

This work was supported by the Hubei Province’s Outstanding Medical Academic Leader program, the Foundation for Innovative Research Team of Hubei Provincial Department of Education (No. T2020025), the general project of Hubei Provincial Department of Education (No. B2021160), Innovative Research Program for Graduates of Hubei University of Medicine (grant no. YC2022042, YC2022037), and the Key Discipline Project of Hubei University of Medicine.

## Conflict of interest

The authors declare that the research was conducted in the absence of any commercial or financial relationships that could be construed as a potential conflict of interest.

## Publisher’s note

All claims expressed in this article are solely those of the authors and do not necessarily represent those of their affiliated organizations, or those of the publisher, the editors and the reviewers. Any product that may be evaluated in this article, or claim that may be made by its manufacturer, is not guaranteed or endorsed by the publisher.
